# *Lactobacillus plantarum* reverse diabetes-induced Fmo3 and ICAM expression in mice through enteric dysbiosis-related c-Jun NH2-terminal kinase pathways

**DOI:** 10.1371/journal.pone.0196511

**Published:** 2018-05-31

**Authors:** Wen-Chung Liu, Ming-Chieh Yang, Ying-Ying Wu, Pei-Hsuan Chen, Ching-Mei Hsu, Lee-Wei Chen

**Affiliations:** 1 Department of Surgery, Kaohsiung Veterans General Hospital, Kaohsiung, Taiwan; 2 School of Medicine, National Yang-Ming University, Taipei, Taiwan; 3 Department of Biological Sciences, National Sun Yat-Sen University, Kaohsiung, Taiwan; 4 Institute of Emergency and Critical Care Medicine, National Yang-Ming University, Taipei, Taiwan; Stellenbosch University, SOUTH AFRICA

## Abstract

Diabetes mellitus (DM) is characterized by increased fatality associated with the atherogenetic process. Circulating trimethylamine-N-oxide (TMAO) levels are closely associated with atherosclerosis. The flavin mono-oxygenase family (Fmo) members oxidize trimethylamine (TMA) to TMAO. The effect and the regulatory mechanism of intestinal microflora on diabetes-induced Fmo3 and intercellular adhesion molecule (ICAM) expression were examined in streptozotocin-induced diabetic mice (STZDM) and Akita mice (C57BL/6J-Ins2^Akita^). STZDM-JNK1^-/-^ and Ins2^Akita^-JNK1^-/-^ mice were produced and used to study the role of pJNK in the regulatory mechanisms. Diabetic mice exhibited decreased *Lactobacilli* growth and reactive oxygen species (ROS) production in the intestinal mucosa; increased levels of pJNK and iNOS proteins in the intestinal mucosa; increased levels of serum nitrate, IL-1β, and TNF-α expression in Kupffer cells; increased Fmo3 expression in the liver; and increased ICAM expression in the aorta. Reversal of diabetes-induced enteric dysbiosis by prebiotic (FOS) or probiotic (dead *L*. *plantarum*) treatment decreased diabetes-induced pJNK and iNOS expression in the intestine, Fmo3 expression in the liver, IL-1β expression in Kupffer cells, and ICAM expression in the aorta and liver. Ins2^Akita^-JNK1^-/-^ and STZDM-JNK1^-/-^ mice demonstrated decreased levels of serum NO, IL-1β expression in Kupffer cells, Fmo3 expression in the liver, and ICAM expression in the aorta. GF mice cohoused with DM mice demonstrated an increase in ICAM expression in the liver. In conclusion, diabetes induced the expression of both Fmo3 and ICAM expression and possible vascular impairment through enteric dysbiosis. Diabetes-induced Fmo3 and ICAM expression could be reversed by pJNK inhibition or by correcting enteric dysbiosis.

## Introduction

Diabetes mellitus (DM) is a metabolic disorder characterized by increased mortality rates and largely involved in the atherogenetic process [1]. DM induces vascular dysfunction through several processes such as hyperlipidemia, hyperinsulinemia, hyperglycemia, insulin resistance, and hyperhomocysteinemia [2]. Intercellular adhesion molecule 1 (ICAM-1), cluster of differentiation-146 (CD146), and vascular cell adhesion molecule 1 (VCAM-1) are largely expressed by endothelial cells, and promote the adhesion and transmigration of immune cells, leading to inflammation. Endovascular inflammation plays an important role in diabetes-mediating adverse effects to the vascular components [3]. The relationship between commensal microflora and diabetes-induced endothelial inflammation and vascular impairment has not yet been clarified. Circulating trimethylamine-N-oxide (TMAO) levels are closely associated with atherosclerosis. The flavin mono-oxygenase family members Fmo1 and Fmo3 oxidize trimethylamine (TMA), originated from gut flora metabolism of choline, to TMAO. Fmo3 has been recognized as a strong candidate responsible for the conversion of TMA to TMAO [4]. However, the effect of diabetes on the regulatory mechanism of intestinal microflora on Fmo3 has not yet been well characterized.

The intestinal tract formed a primary physical barrier between the microflora and internal host tissue and responds to the mucosal innate system through the commensal microflora [5]. Paneth cells are critical contributors to restrain bacterial penetration and to the small intestinal antimicrobial barrier through production and release of antimicrobial peptides and proteins such as enteric CRS (cryptdin-related sequences) peptides, α-defensins, lysozyme, RegIIIβ, RegIIIγ, CRP-ductin, and RELMβ (resistin-like molecules β) [6]. Different mouse models demonstrated that inflammasome-deficiency-associated changes in the configuration of the gut microbiota are closely associated with increased hepatic steatosis and inflammation through influx of TLR4 and TLR9 agonists into the portal circulation system, leading to increased hepatic tumor-necrosis factor (TNF-α) expression that induce chronic hepatic inflammation, non-alcoholic steatohepatitis (NASH) [7]. Prebiotics are non-digestible short-chain oligosaccharides which access colon and are fermented to change the GI environment (acid pH and increased shot-chain fatty acid) to selectively induce the growth of several commensal bacteria such as *bifidocateria* and *lactobacillus* [8].

At normal levels, nitric oxide (NO) is an important mediator of intestinal cell and barrier function [9]. When NO is present redundant, however, the result is barrier dysfunction [10]. It is commonly recognized that DM decreases endothelial nitric oxide synthase (eNOS) activity as well as increases the production of reactive oxygen species (ROS), thus resulting in decreased nitric oxide (NO) bioavailability and the following pro-atherogenetic alterations (3). However, the involvement of reactive oxygen species (ROS) production and iNOS expression in the intestinal mucosa in diabetes-induced vascular dysfunction is also still not clear. The primary objective of this study was to determine the effect of diabetes on ICAM and Fmo3 expression in the liver. The secondary objective was to determine whether feeding with dLac or fructooligosaccharides (FOS) could reverse the stimulatory effects of diabetes on Fmo3 and ICAM expression. The third objective was to determine whether JNK inhibition could decrease NO production and diabetes-induced Fmo3 and ICAM expression.

## Materials and methods

### Animals and treatments

Specific pathogen-free (SPF) (total n = 280) and germ-free C57BL/6J mice (total n = 60) were purchased from the National Laboratory Breeding and Research Center (NLBRC, Taipei, Taiwan). Ins2-Akita (Ins2^Akita^ mutation mutant) mice (C57BL/6J background) (total n = 124) were purchased from the Jackson Laboratory (Bar Harbor, ME). JNK1^-/-^ (c-Jun N-terminal kinases 1 knockout) mice (C57BL/6J background) (total n = 72) generated from the same background were transferred from Dr. Karin’s laboratory (University of California, San Diego, CA, USA). The Ins2^Akita^ mutation causes a single amino acid substitution in the insulin 2 gene that causes misfolding of the insulin protein[11]. Male mice heterozygous for this mutation have progressive loss of β-cell function and significant hyperglycemia, as early as 4 weeks of age. We obtained Ins2^Akita^-JNK1^-/-^ mice (total n = 72) by crossbred Ins2^Akita^ mice with JNK1^-/-^ mice. To develop a diabetic mouse model, male C57BL/6 or JNK1^-/-^ mice were administered one intraperitoneal (i.p.) injection of streptozotocin (STZ, Sigma-Aldrich) to induce the death of pancreatic β cells. Mice with two consecutive readings of blood glucose >250 mg/dl were recognized diabetic. All mice had ad libitum access to water and food and were fed a standard laboratory diet (1324 TPF; Atromin; Large Germany; 11.9 kJ/g, 19% crude protein, 4% crude fat, 6% crude fiber).

### Ethics statement

The Institutional Animal Care and Use Committee of Kaohsiung Veterans General Hospital has approved this study (Permit Number: 2015-2018-A007), and animal experiments were performed in agreement with Animal Experimentation Regulations of Kaohsiung Veterans General Hospital. All efforts were made to reduce suffering of animals. Animals were checked every 6 hours for signs of distress and endpoints. Specific criteria used to decide when the animals should be euthanized were in accordance with Remick lab report [12]. Mice were euthanized with avertin (250 mg/kg, Sigma) when they were found in a moribund state as identified by inability to maintain upright with or without labored breathing and cyanosis. No mortality occurred outside of planned euthanasia or humane endpoints.

### FOS or dead L. plantarum feeding

To investigate the effects of intestinal dysbiosis on diabetes-induced Fmo3 and intercellular adhesion molecule (ICAM) expression, prebiotic (FOS, 250 mg/day; Sigma-Aldrich) was given in drinking water to mice for 6 days (n = 50 in each group). To verify that the mechanisms for improvement and prevention by FOS supplementation are through enhancing specific groups of intestinal commensal microbiota (*Lactobacillus*) to reduce FMO3 and ICAM expression, diabetic mice were fed with dead *Lactobacillus plantarum* (2×10^8^ CFU/ml)[13] in drinking water for 6 days (n = 50 in each group). *Lactobacillus plantarum* were killed by heating at 63°C for 30 minutes. The water with FOS or bacteria was refreshed every day. The dead bacteria were completely suspended in the drinking water. There was no precipitation of bacteria in the bottle. The control group received drinking water without the supplementation of prebiotic or dead *Lactobacillus plantarum*.

### 16S rRNA gene sequencing and analysis

Genomic DNA of intestine was extracted from different groups in triplicate and amplified a portion of the V2 region of the 16S rRNA gene of bacteria using barcoded primers, followed by high-throughput sequencing of amplicons. We generated approximately 20,000 high quality sequences per sample. Sequences were demultiplexed and analyzed with the QIIME (Quantitative Insights Into Microbial Ecology) software package [14].

### Bacterial DNA extraction and quantitative real-time PCR

Bacterial genomic DNA was purified from terminal ileum using the Qiagen DNA stool kit according to the manufacturer’s directions. The number of specific bacterial groups was calculated by using StepOnePlus™ Real-Time PCR System (Applied Biosystems 7300) [15].

### Western immunoblots

The Reg3β, iNOS, and ICAM protein expression in the intestinal mucosa were identified by mouse monoclonal antibodies (R&D Systems). JNK and pJNK was identified by mouse monoclonal antibodies (Santa Cruz Biotechnology Inc.). The harvested tissues were weighed and homogenized in protein extraction buffer (Sigma) containing proteinase inhibitor cocktail (Roche). The homogenized samples were subjected to SDS-PAGE at 50 to 100 V for 2 hr. The proteins were transferred onto the nitrocellulose membrane. The membrane was blocked with 5% non-fat milk in TBST buffer (10 mM Tris-HCl, pH 7.5, 150 mM NaCl and 1.2% Tween 20) for 1 hr and incubated with specific primary antibodies at room temperature for 1 hr. After immunoblotting with the primary antibodies, the membranes were washed with TBST buffer and incubated with the secondary antibodies. The membranes were washed with TBST buffer and the protein bands were identified by enhanced chemiluminescence (ECL) detection reagent (Millipore).

### ROS levels in the intestinal mucosa

The levels of ROS in the intestinal mucosa were analyzed by 10 mM DCFDA fluorescent dye (Sigma), which was added into the suspension of intestinal mucosa for cultivation. DCFDA is deacetylated by cellular esterases to a non-fluorescent compound that is later oxidized by ROS into 2ʹ7ʹ–dichlorofluorescein (DCF). DCF is identified by fluorescence spectroscopy with excitation and emission spectra of 495 nm and 529 nm, respectively [16].

### RNA isolation and quantitative real-time polymerase chain reaction (qRT-PCR)

Total RNA was purified from mouse liver samples using total RNA Miniprep Purification Kits (GeneMark) according to the manufacturer’s instructions. Total RNA was then reverse-transcribed into cDNA using a RT kit (Invitrogen, Carlsbad, CA). Primer pairs were synthesized by Integrated DNA Technologies (Coralville, IA) and are as follow: Fmo3 forward: 50-GGA AGAGTTGGT GAAGAC CG-30, reverse: 50-CCC ACA TGC TTT GAG AGG AG-30. For the real-time PCR assay, 200 ng of the cDNA template was added to 20 μl of mixture containing 12.5 μl of 2X KAPA SYBR^®^ FAST qPCR Master Mix (Kapa Biosystems), 2.5 μl of each sense and anti-sense primers (25 μM) and 5 μl of sterile water. The amplification was performed in a StepOnePlus™ Real-Time PCR System (Applied Biosystems 7300).

### Kupffer cell purification

The liver was perfused *in situ* through the portal vein with Ca^2+^- and Mg^2+^-free phosphate-buffered saline containing 10 mM ethylenediaminetetraacetic acid at 37°C for 5 minutes. Subsequently perfusion was performed with HBSS containing 0.1% collagenase IV (Sigma) at 37°C for 5 minutes. After digestion, the liver was excised and the suspension was filtered. The filtrate was centrifuged twice at 50×g at 4°C for 1 minute. The supernatant was harvested and centrifuged at 300g for 5minutes, and the pellet was resuspended with buffer. The cell suspension was then layered on top of a density cushion of 30%/60% discontinuous Percoll (Pharmacia) and centrifuged at 900×g for 15 minutes to obtain the Kupffer cell fraction, followed by washing with the buffer again [17].

### Expression of IL-1β and IL-6 in Kupffer cells

The total RNAs were extracted from Kupffer cells with the Miniprep Purification Kit (GeneMark). Real-time polymerase chain reaction was performed with the SYBR Green PCR Master Mix and ABI PRISM 7700 Sequence Detection Systems (Applied Biosystems, Foster City, CA) according to the manufacturer’s suggested protocol. Sets of IL-6 and IL-1β primers were designed according to those genes documented in GenBank [18].

### Griess reagent assay

A 100 μl of serum was mixed with 40 μl of Griess reagent in each well of the ELISA plate. The mixture was incubated at room temperature for 20 min in the dark and measured for the absorbance at 550 nm. Nitrates are measured as indication of NO production. The concentration of NO in serum was determined as compared to the standard curve.

### Cohousing germ-free mice

Germ-free C57BL/6J mice were produced by the National Laboratory Breeding and Research Center and transferred to our animal facility in a transfer chamber compatible with the isolator. After arrival, a GF mouse was randomly chosen to cohouse with a wild-type mouse or Ins2^Akita^ mouse and kept in a sterile lamina-flow hood for 2 weeks (n = 6 in each group) to examine whether the microbiota of the Ins2^Akita^ could change the antibacterial protein expression of the GF mice.

### Statistical analysis

All immunoblotting and electrophoretic mobility shift assays were assessed by densitometric scanning. All data are analyzed by one-way analysis of variance (ANOVA), followed by Tukey’s Multiple Comparison Test. All values in the figures and text were expressed as mean ± standard error of the mean, and P values of less than 0.05 are considered to be statistically significant.

## Results

### Diabetes induces enteric dysbiosis and FOS feeding reverses it

**Fig 1:**
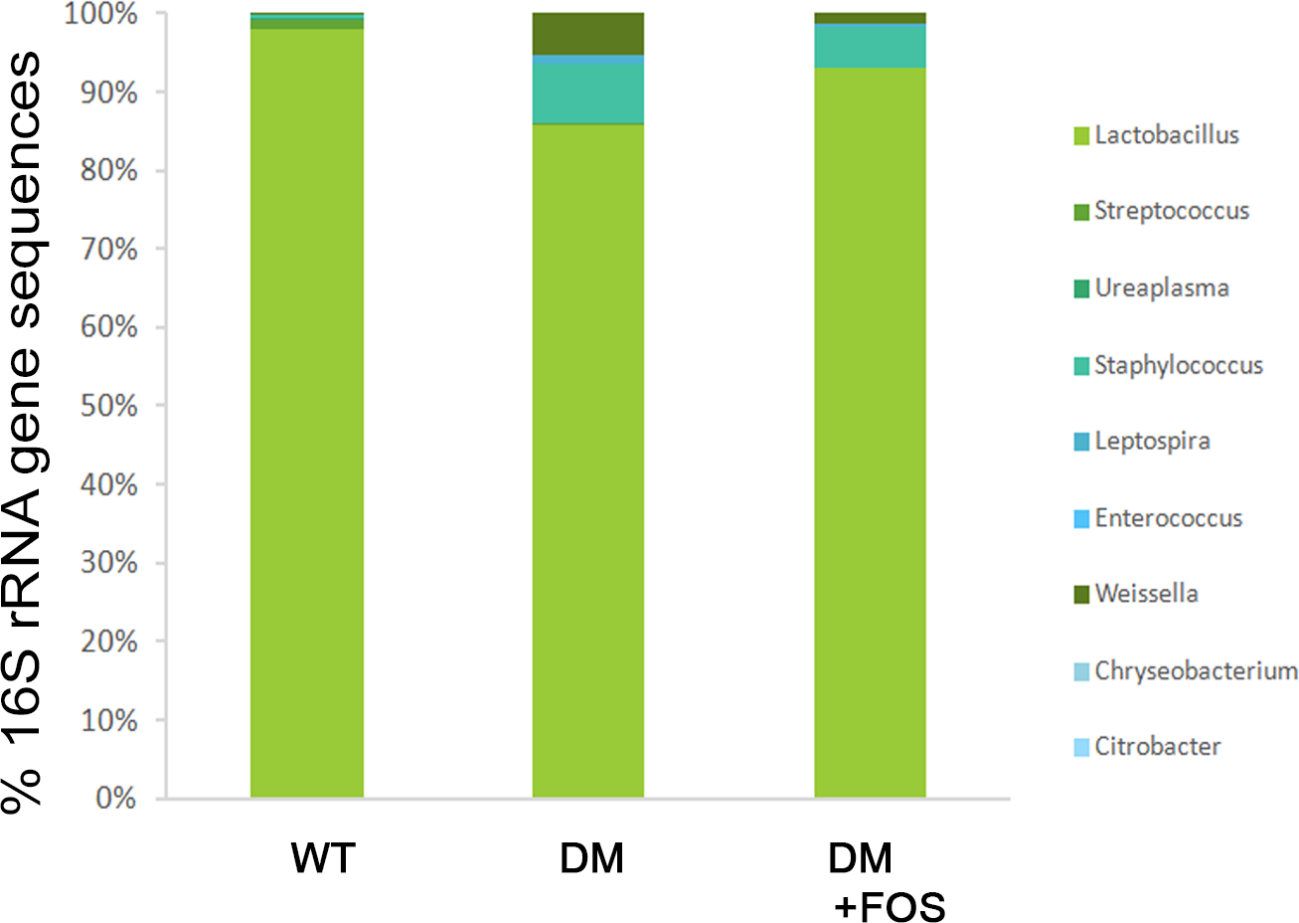
Diabetes induced enteric dysbiosis and FOS feeding reversed it. Relative abundance of bacteria across difference groups, as indicated by 16S rRNA gene sequencing. Values represent the mean abundance of Genus found at >1% relative abundance in at least one sample. High-throughput 16S rRNA gene sequencing revealed that *Lactobacillus* was decreased in DM group compared with WT group and FOS feeding significantly increased *Lactobacillus* in the intestine in STZ-DM mice. STZ, streptozotocin; FOS, fructooligosaccharides; DM, diabetes mellitus. n = 3/group.

FOS or dead L. plantarum feeding did not change body weight or blood glucose levels in STZ-DM mice S1 Table. To determine whether enteric dysbiosis plays an important role in diabetes-induced Fmo3 and ICAM expression, genomic DNA of the guts was harvested from different groups and examined by high-throughput sequencing of amplicons. Next, STZ-DM mice were fed with a prebiotic, FOS, to reverse the effect of enteric dysbiosis in diabetes. FOS supplementation did not change the body weight or blood glucose levels in STZ-DM mice. High-throughput 16S rRNA gene sequencing revealed that *Lactobacillus* growth was decreased in the DM group compared with that in the WT group, and FOS feeding significantly increased the *Lactobacillus* growth in the intestine of STZ-DM mice (Fig 1).

### Diabetes reduces Reg3β protein expression in the intestinal mucosa and FOS or dead L. plantarum feeding reverses it

**Fig 2:**
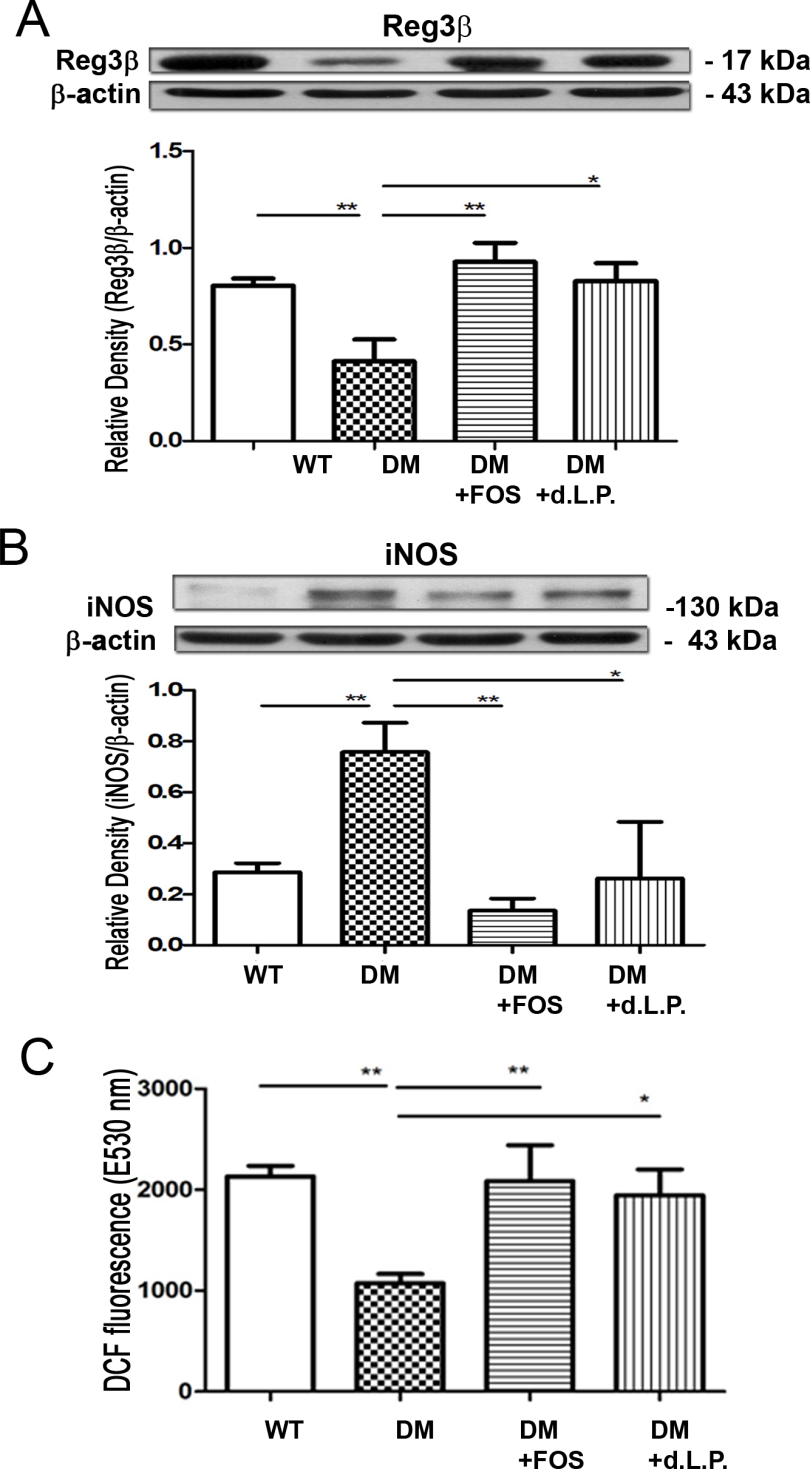
Diabetes induced iNOS expression and reduced Reg3β expression and ROS levels of the intestinal mucosa. (A) Reg3β protein expression in the intestinal mucosa was examined in STZDM mice with Western blotting. FOS or dead *L*. *plantarum* feeding increased Reg3β protein expression in the intestinal mucosa compared with that of STZDM mice. (B) STZDM mice demonstrated a significant increase of iNOS protein expression in the intestinal mucosa compared with WT group, and FOS or dead *L*. *plantarum* feeding decreased it. (C) STZDM mice induced a decrease of DCFDA levels in the intestinal mucosa compared with WT group, and FOS or dead *L*. *plantarum* feeding reversed them. The levels of ROS in the intestinal mucosa were analyzed by DCFDA fluorescent dye, which was added into the suspension of intestinal mucosa for the cultivation. DCFDA is oxidized by ROS into 2ʹ7ʹ–dichlorofluorescein (DCF). DCF is detected by fluorescence spectroscopy with excitation and emission spectra of 495 nm and 529 nm, respectively. STZ, streptozotocin; DM, diabetes mellitus; iNOS, inducible nitric oxide synthase; DCFDA, 2’,7’–dichlorofluorescin diacetate; FOS, fructooligosaccharides; dLac, dead *L*. *plantarum*. *P<0.05, **P<0.01. n = 5/group.

Reg3β is one of the antimicrobial proteins of the intestinal mucosa that contribute to limiting the bacterial penetration and to the small intestinal antimicrobial barrier. The protein expression of mucosal Reg3β was significantly decreased by 49% in STZ-DM mice compared with that in WT mice (Fig 2A). FOS and dead *L*. *plantarum* feeding significantly increased the Reg3β protein expression in the intestinal mucosa to 225% and 207%, respectively, compared with that in STZ-DM mice (Fig 2A).

### Diabetes induces iNOS protein expression in the intestinal mucosa and FOS or dead L. plantarum feeding reverses it

To assess the effect of diabetes on the production of ROS and NO in the intestinal mucosa, we examined iNOS protein expression in the intestinal mucosa of STZ-DM mice. STZ-DM mice demonstrated a significant two-and-half-fold (264%) increase in iNOS protein expression in the intestinal mucosa compared with that in the control group (Fig 2B). FOS and dead *L*. *plantarum* feeding significantly decreased 82% and 65% of iNOS expression in the intestinal mucosa of STZ-DM mice, respectively (Fig 2B). These findings indicate that diabetes induces iNOS protein expression in the intestinal mucosa and FOS or dead *L*. *plantarum* feeding reverses it.

### Diabetes decreases ROS levels in the intestinal mucosa and FOS or dead L. plantarum feeding restores them

The effect of diabetes on ROS production in the intestinal mucosa was assessed by examining the DCFDA levels in the intestinal mucosa of STZ-DM mice. STZ-DM mice demonstrated a significant 52% decrease in DCFDA levels in the intestinal mucosa compared with that in the control group (Fig 2C). FOS or dead *L*. *plantarum* feeding significantly increased the DCFDA levels by two-fold in the intestinal mucosa of STZ-DM mice (Fig 2C) compared with that in STZ-DM mice. These results show that diabetes decreases the production of ROS in the intestinal mucosa and FOS or dead *L*. *plantarum* feeding restores it.

### Diabetes induces Fmo3 expression in the liver and FOS or dead *L*. *plantarum* feeding reverses it

**Fig 3:**
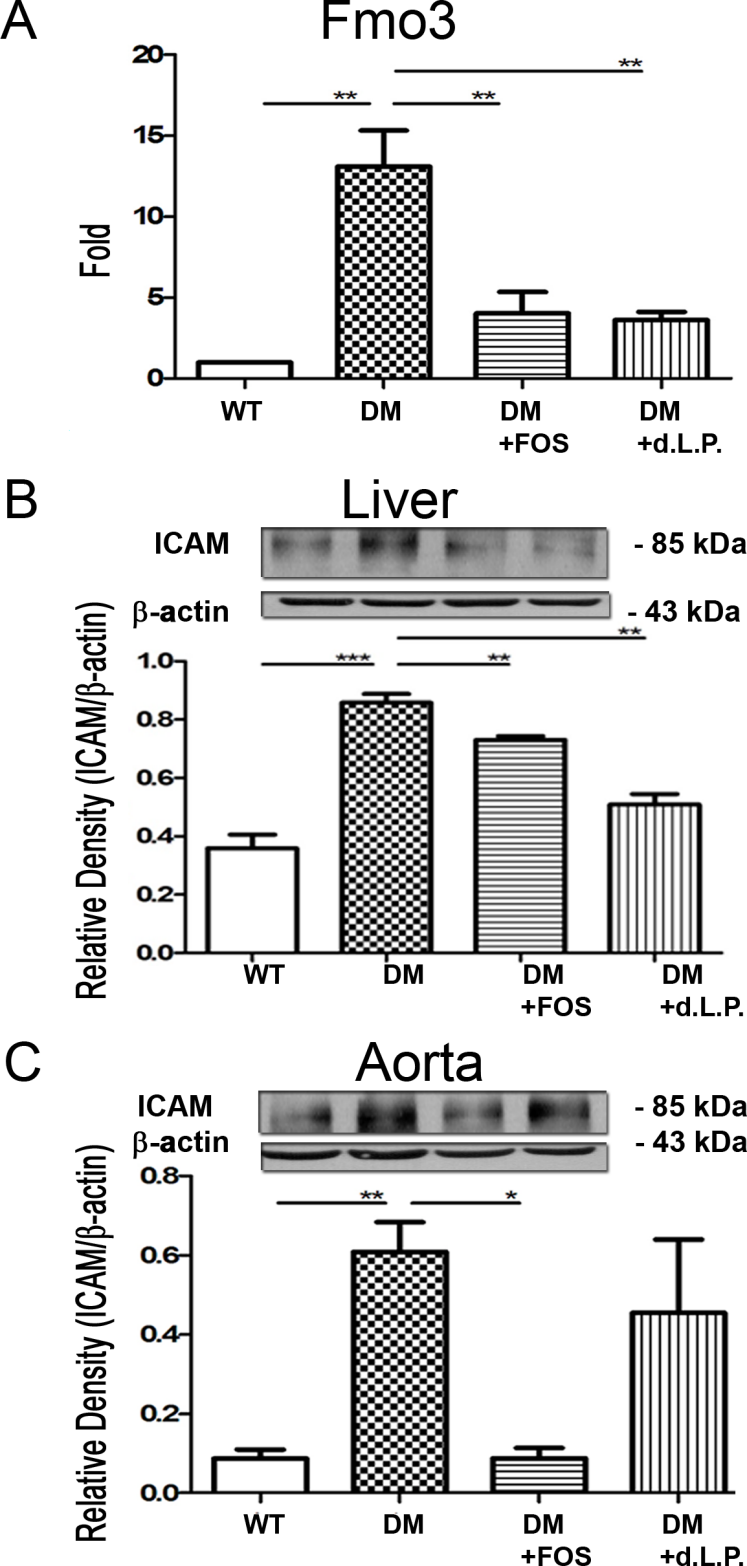
Diabetes induced Fmo3 in the liver and ICAM expression in the liver and aorta. (A) Fmo3 mRNA expression in the liver was examined with real time PCR in STZDM mice. Diabetes induced a significant increase of Fmo3 expression in the liver in STZDM mice compared with that in the WT group. FOS or dead *L*. *plantarum* feeding decreased FMO3 mRNA expression in the liver in STZDM mice. (B) ICAM protein expression in liver was examined with Western blotting in STZDM mice. Diabetes induced a significant three-fold increase of ICAM protein expression in the liver in STZDM mice compared with that in the WT group. FOS or dead *L*. *plantarum* feeding decreased ICAM expression in the liver. (C) ICAM protein expression in the aorta was examined with Western blotting in STZDM mice. Diabetes induced an increase in ICAM expression in the aorta in STZDM mice compared with that in the WT group. FOS feeding decreased ICAM expression in the aorta in STZDM mice. ICAM, intercellular adhesion molecule; Fmo, flavin mono-oxygenase; STZ, streptozotocin; DM, diabetes mellitus; FOS, fructooligosaccharides; dLac, dead *L*. *plantarum*. *P<0.05, **P<0.01, ***P<0.001. n = 6/group.

Increased Fmo3 expression in the liver is closely related to metabolic syndrome and cholestasis in diabetes [19]. The effect of diabetes on Fmo3 expression in the liver was assessed by examining Fmo3 mRNA expression in the liver using real-time PCR in STZ-DM mice. Diabetes induced a significant increase in Fmo3 expression in the liver of STZ-DM mice compared with that in the control group (Fig 3A). FOS or dead *L*. *plantarum* feeding significantly decreased Fmo3 expression in the liver of STZ-DM mice. These results suggest that diabetes induces Fmo3 expression in the liver and FOS or dead *L*. *plantarum* feeding reverses it.

### Diabetes induces ICAM expression in the liver and FOS or dead *L*. *plantarum* feeding reverses it

To determine the changes in ICAM expression in the liver in diabetes, the ICAM protein expression in the liver of STZ-DM mice was examined by Western blotting. Diabetes induced a significant three-fold increase in ICAM protein expression in the liver of STZ-DM mice compared with that in the control group (Fig 3B). FOS or dead *L*. *plantarum* feeding significantly decreased 20% and 48% of ICAM expression, respectively, in the liver compared with that in STZ-DM mice. These observations indicate that diabetes induces ICAM expression in the liver, and FOS or dead *L*. *plantarum* feeding reverses it.

### Diabetes induces ICAM expression in the aorta and FOS feeding reverses it

The effect of diabetes on ICAM expression in the aorta was examined by analyzing the ICAM protein expression in the aorta of STZ-DM mice. Diabetes induced a significant seven-fold increase in ICAM expression in the aorta of STZ-DM mice compared with that in the control group (Fig 3C). FOS feeding significantly decreased 75% of ICAM expression in the aorta of STZ-DM mice, whereas dead *L*. *plantarum* feeding did not decrease the ICAM expression in the aorta of STZ-DM mice. These data indicate that diabetes induces ICAM expression in the aorta and FOS feeding reverses it.

### Ins2^Akita^ mice demonstrate decreased Reg3β protein expression in the intestinal mucosa and FOS or dead *L*. *plantarum* feeding increases it

**Fig 4:**
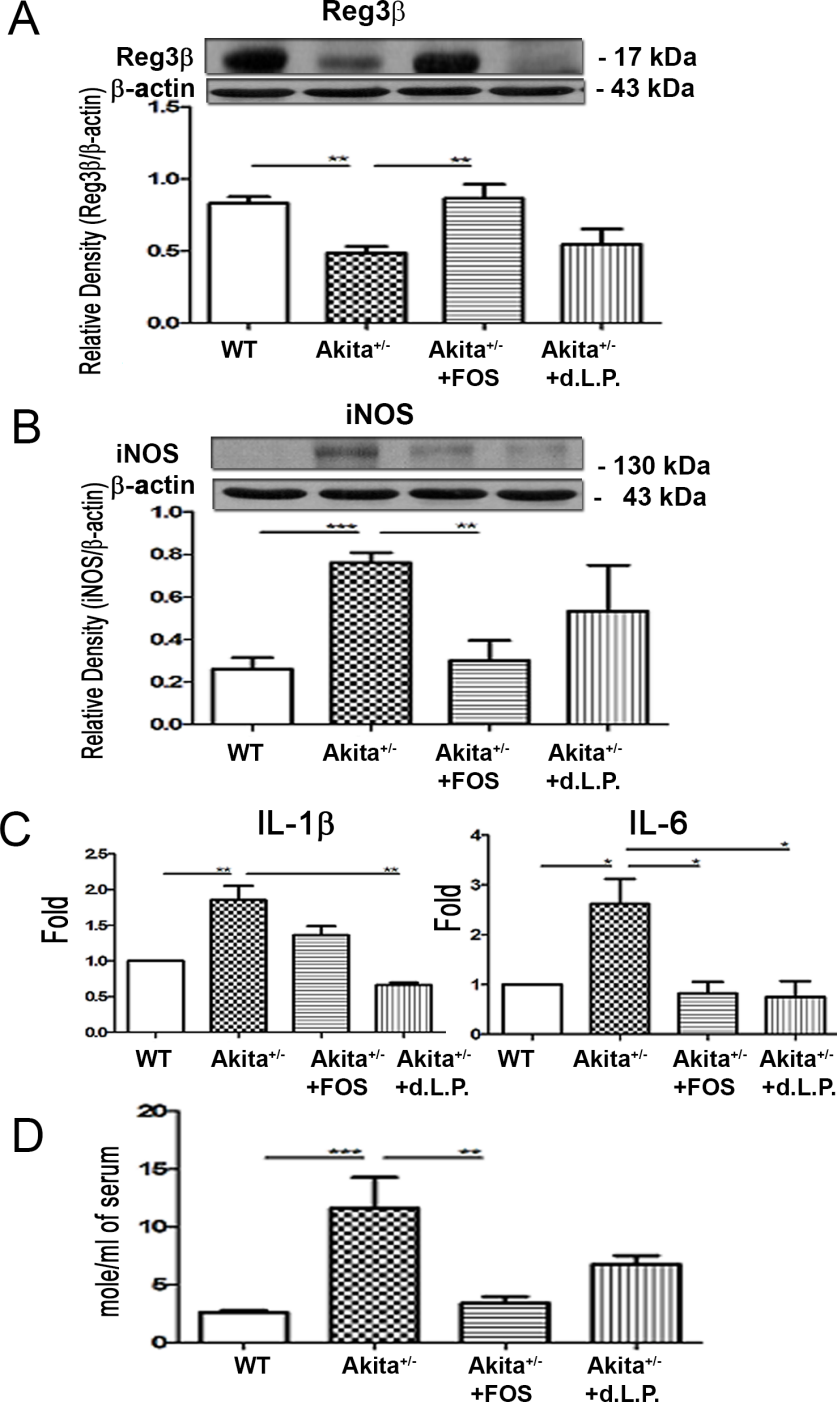
Ins2^Akita^ mice demonstrated increased iNOS expression in the intestinal mucosa, IL-1β and IL-6 expression in Kupffer cells, and serum NO levels and FOS or dead *L*. *plantarum* feeding increased it. (A) Reg3β protein expression in the intestinal mucosa was examined in STZDM mice with Western blotting. The protein expression of mucosal Reg3β was decreased in Ins2^Akita^ mice. FOS feeding increased Reg3β protein expression in the intestinal mucosa. (B) Ins2^Akita^ mice demonstrated an increase of iNOS protein expression in the intestinal mucosa compared with that in WT group. FOS feeding decreased the iNOS expression in the intestinal mucosa in Ins2^Akita^ mice. (C) Ins2^Akita^ mice demonstrated a significant increase of IL-1β and IL-6 expression in Kupffer cells compared with that in the WT mice. FOS feeding decreased IL-6 expression in Kupffer cells in Ins2^Akita^ mice. Dead *L*. *plantarum* feeding decreased IL-1β and IL-6 expression in Kupffer cells in Ins2^Akita^ mice. Kupffer cells were purified from liver. The total RNAs were extracted from Kupffer cells using the Miniprep Purification Kit (GeneMark). Real-time polymerase chain reaction was performed with the SYBR Green PCR Master Mix and ABI PRISM 7700 Sequence Detection Systems (Applied Biosystems, Foster City, CA). (D) Ins2^Akita^ mice demonstrated a three-fold increase of serum NO levels compared with that in the WT mice. FOS treatment induced a decrease of serum NO levels in Ins2^Akita^ mice. The concentration of NO in serum was examined with Griess reagent. iNOS, inducible nitric oxide synthase; NO, nitric oxide; STZ, streptozotocin; DM, diabetes mellitus; FOS, fructooligosaccharides; dLac, dead *L*. *plantarum*. *, *P <* 0.05; **, *P <* 0.01; ***, *<* 0.001. n = 6/group.

To further examine whether FOS or dead *L*. *plantarum* feeding could reverse Reg3β protein expression in the intestinal mucosa in diabetes, we examined Reg3β protein expression in the intestinal mucosa of Ins2^Akita^ mice. The protein expression of mucosal Reg3β was significantly decreased by 62% in Ins2^Akita^ mice compared with that in WT mice (Fig 4A). FOS feeding significantly increased Reg3β protein expression in the intestinal mucosa to 210% in Ins2^Akita^ mice compared with that in Ins2^Akita^ mice (Fig 4A). These results corroborate that diabetes decreases Reg3β protein expression in the intestinal mucosa and FOS feeding restores it.

### Ins2^Akita^ mice demonstrate increased iNOS protein expression in the intestinal mucosa and FOS feeding decreases it

The impact diabetes on iNOS expression in the intestinal mucosa was analyzed by examining the iNOS protein expression in the intestinal mucosa of Ins2^Akita^ mice. Ins2^Akita^ mice demonstrated a significant three-fold increase in iNOS protein expression in the intestinal mucosa compared with that in the WT group (Fig 4B). FOS feeding significantly decreased the iNOS expression in the intestinal mucosa of Ins2^Akita^ mice. These results further prove that diabetes induces iNOS protein expression in the intestinal mucosa and FOS feeding reverses it.

### Ins2^Akita^ mice demonstrate increased IL-1β and IL-6 expression in Kupffer cells and FOS or dead *L*. *plantarum* feeding reverses the expression

Increased cytokine production in Kupffer cells is closely related to hepatocyte injury and cholestasis [19]. To examine the changes of inflammatory cytokines in Kupffer cells in diabetes, the expression levels of IL-1β and IL-6 expression in Kupffer cells were analyzed in Ins2^Akita^ mice. There mice demonstrated a significant increase in IL-1β and IL-6 expression in Kupffer cells compared with that in WT mice (Fig 4C). FOS feeding significantly decreased the IL-6 expression in Kupffer cells in Ins2^Akita^ mice. Dead *L*. *plantarum* feeding significantly decreased IL-1β and IL-6 expression in Kupffer cells of Ins2^Akita^ mice. These findings show that diabetes induces cytokine expression in Kupffer cells, and FOS or dead *L*. *plantarum* feeding reverses the expression.

### FOS feeding reverses diabetes-induced serum NO levels in Ins2^Akita^ mice

The changes in NO levels in diabetes were assessed by examining the serum NO levels in Ins2^Akita^ mice. These mice demonstrated a significant three-fold increase in serum NO levels compared with that in WT mice. FOS treatment induced a significant 57% decrease in serum NO levels in Ins2^Akita^ mice (Fig 4D). These data suggest that diabetes induces serum NO levels, and FOS treatment reverses these levels.

### FOS or dead *L*. *plantarum* feeding reverses diabetes-induced Fmo3 expression in the liver of Ins2^Akita^ mice

**Fig 5:**
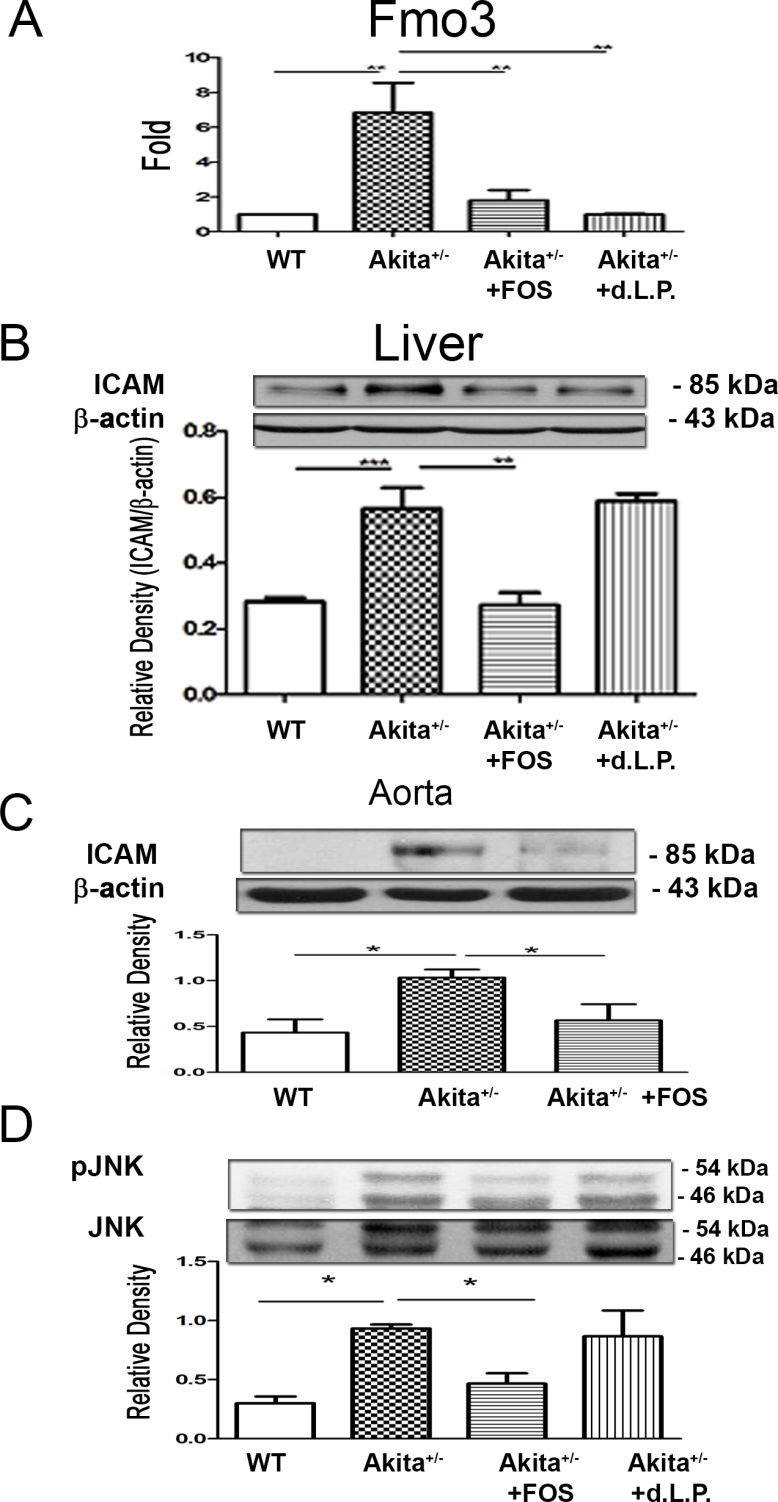
FOS or dead *L*. *plantarum* feeding reversed diabetes-induced pJNK in gut, Fmo3 expression in the liver, and ICAM expression in the liver and aorta in Ins2^Akita^ mice. **(A)** Ins2^Akita^ mice demonstrated a four-fold increase of Fmo3 expression in the liver compared with that in the control group. FOS or dead *L*. *plantarum* feeding decreased FMO3 expression in the liver in Ins2^Akita^ mice. (B) Ins2^Akita^ mice demonstrated a two-fold increase of ICAM expression in the liver compared with that in WT mice. FOS feeding decreased ICAM expression in the liver in Ins2^Akita^ mice. (C) Ins2^Akita^ mice demonstrated a significant increase of ICAM expression in the aorta compared with that in WT mice. FOS feeding decreased ICAM expression in the aorta in Ins2^Akita^ mice. (D) Ins2^Akita^ mice demonstrated a significant increase of pJNK expression in the intestinal mucosa compared with that in WT mice. FOS feeding decreased pJNK expression in the intestinal mucosa in Ins2^Akita^ mice. ICAM, intercellular adhesion molecule; pJNK, phosphor c-Jun NH2-terminal kinase; Fmo, flavin mono-oxygenase; DM, diabetes mellitus; FOS, fructooligosaccharides; dLac, dead *L*. *plantarum*. *, *P* < 0.05; **, *P* < 0.01; ***, < 0.001. n = 6/group.

To further corroborate the changes in Fmo3 expression in the liver in diabetes, Fmo3 expression in the liver of Ins2^Akita^ mice was examined. We found that Ins2^Akita^ mice demonstrated a significant four-fold increase in Fmo3 expression in the liver compared with that in the control group (Fig 5A). FOS or dead *L*. *plantarum* feeding significantly decreased the Fmo3 expression in the liver of Ins2^Akita^ mice. These results further prove that diabetes induces Fmo3 expression in the liver and FOS or dead *L*. *plantarum* feeding reverses it.

### FOS feeding reverses diabetes-induced ICAM expression in the liver of Ins2^Akita^ mice

The changes in ICAM expression in the liver in diabetes were assessed by examining the ICAM expression in the liver of Ins2^Akita^ mice. These mice demonstrated a significant two-fold increase in ICAM expression in the liver compared with that in WT mice (Fig 5B). FOS feeding significantly decreased the ICAM protein expression in the liver of Ins2^Akita^ mice. These observations indicate that diabetes induces ICAM expression in the liver and FOS feeding reverses it.

### FOS feeding reverses diabetes-induced ICAM expression in the aorta of Ins2^Akita^ mice

We analyzed the changes in ICAM expression in the aorta of Ins2^Akita^ mice which demonstrated a significant increase in ICAM expression in the aorta compared with that in WT mice (Fig 5C). FOS feeding significantly decreased the ICAM protein expression in the aorta of Ins2^Akita^ mice, indicating that diabetes induces ICAM expression in the aorta and FOS feeding reverses it.

### FOS feeding reverses diabetes-induced pJNK expression in intestinal mucosa of Ins2^Akita^ mice

To examine the involvement of pJNK in the intestinal mucosa in diabetes, we analyzed pJNK1 protein expression in the intestinal mucosa of Ins2^Akita^ mice. These mice demonstrated a significant increase in pJNK expression in the intestinal mucosa compared with that in WT mice (Fig 5D). FOS feeding significantly decreased the pJNK expression in the intestinal mucosa of Ins2^Akita^ mice, which indicate that diabetes induces pJNK expression in the intestinal mucosa, and FOS feeding reverses it.

### JNK1^-/-^ mice demonstrates decreased JNK protein expression of aorta

**Fig 6:**
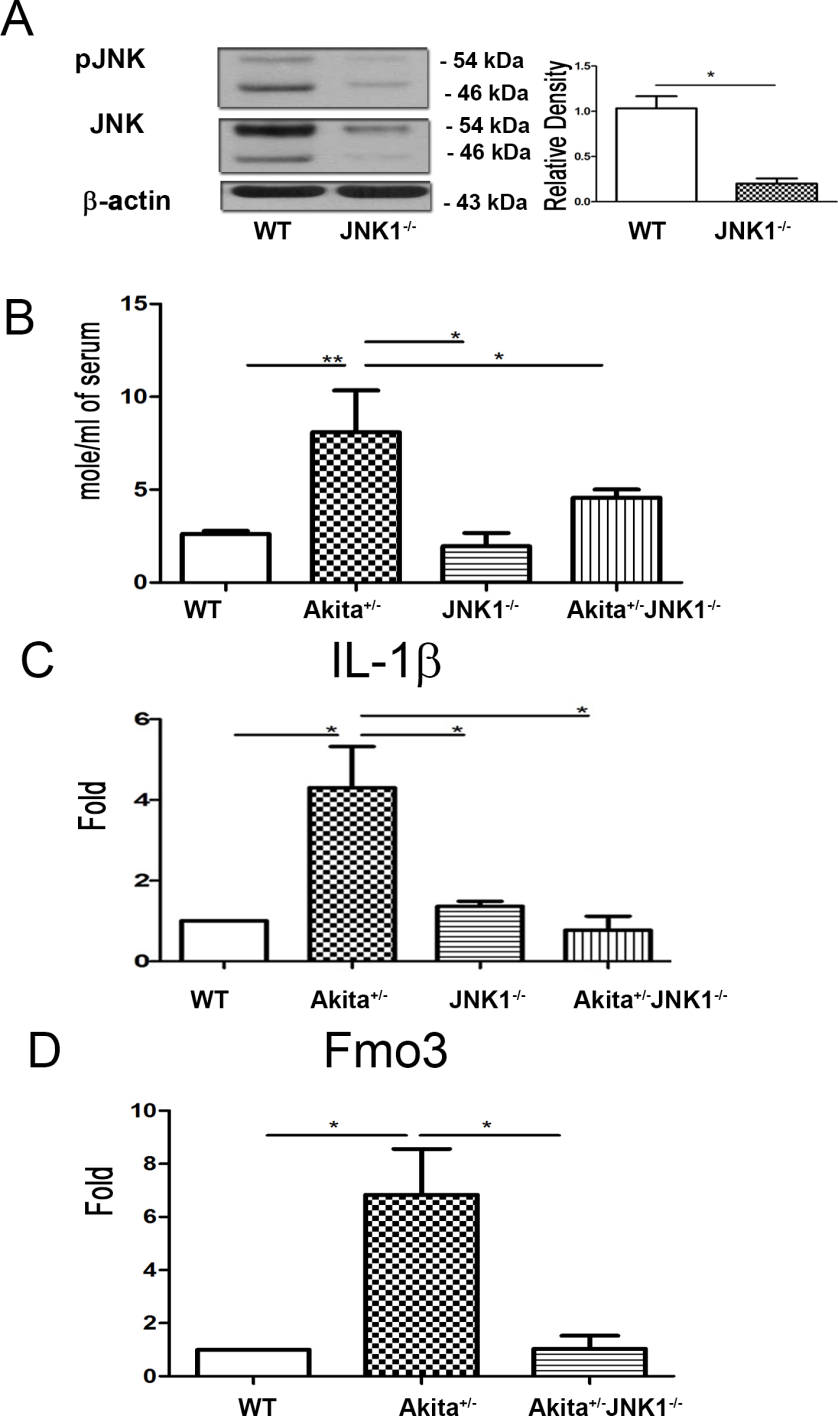
Ins2^Akita^-JNK1^-/-^ mice demonstrated reduction of serum NO levels, IL-1β expression in Kupffer cells, and Fmo3 expression in the liver compared with Ins2^Akita^ mice. (A) JNK1^-/-^ mice demonstrated significantly decreased pJNK and JNK protein expression of aorta compared with WT mice. (B) Ins2^Akita^-JNK1^-/-^ mice demonstrated decreased serum NO levels compared with Ins2^Akita^ mice. (C) Ins2^Akita^-JNK1^-/-^ mice demonstrated a significant decrease of IL-1β expression in Kupffer cells compared with Ins2^Akita^ mice. (D) Ins2^Akita^-JNK1^-/-^ mice demonstrated a significant decrease of Fmo3 expression in the liver compared with that in Ins2^Akita^ mice. JNK, c-Jun NH2-terminal kinase. *, *P* < 0.05; **, *P* < 0.01; ***, < 0.001. n = 6/group.

JNK1^-/-^ mice demonstrated decreased JNK (JNK1 expressed predominantly as p46 JNK with lesser amounts of p54 JNK) and pJNK protein expression of aorta as compared with that in WT mice (Fig 6A).

### Ins2^Akita^-JNK1^-/-^ mice demonstrates decreased serum NO levels

We examined the role of pJNK1 in diabetes-induced serum NO levels by analyzing the serum NO levels in Ins2^Akita^-JNK1^-/-^ mice. These mice demonstrated a significant decrease in serum NO levels compared with that in Ins2^Akita^ mice (Fig 6B). These data further prove that phospho-JNK plays a critical role in diabetes-induced serum NO levels, and pJNK1 inhibition decreases the serum NO levels induced by diabetes.

### Ins2^Akita^-JNK1^-/-^ mice demonstrate decreased IL-1β expression in Kupffer cells

To examine the role of pJNK1 in diabetes-induced activation of Kupffer cells, we analyzed the IL-1β expression in Kupffer cells in Ins2^Akita^-JNK1^-/-^ mice. These mice demonstrated a significant four-fold decrease in IL-1β expression in Kupffer cells compared with that in Ins2^Akita^ mice (Fig 6C). These findings indicate that phospho-JNK1 plays a critical role in diabetes-induced Kupffer cell activation, and pJNK1 inhibition decreases the diabetes-induced activation of Kupffer cells.

### Ins2^Akita^-JNK1^-/-^ mice demonstrate reduction of Fmo3 expression

The role of pJNK1 in diabetes-induced Fmo3 expression in the liver was assessed by analyzing Fmo3 expression in the liver of Ins2^Akita^-JNK1^-/-^ mice. We found that Ins2^Akita^-JNK1^-/-^ mice demonstrated a significant decrease in Fmo3 expression in the liver compared with that in Ins2^Akita^ mice (Fig 6D). These results further prove that phospho-JNK plays a critical role in diabetes-induced Fmo3 expression in the liver, and pJNK1 inhibition decreases the Fmo3 expression induced by diabetes.

### STZDM-JNK1^-/-^ mice demonstrate decreased serum NO levels

**Fig 7:**
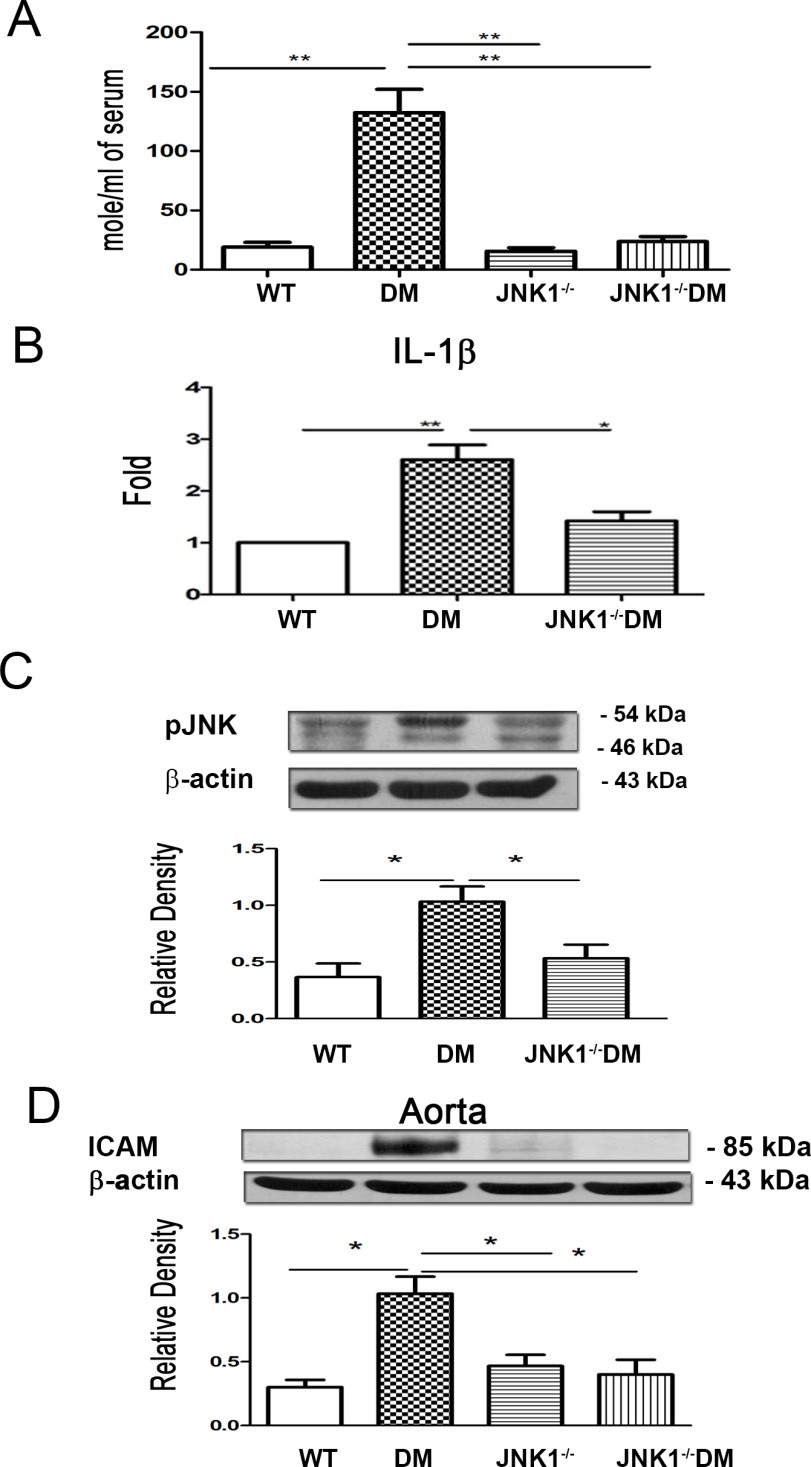
STZDM-JNK1^-/-^ mice demonstrated a significant decrease of serum NO levels, IL-1β expression in Kupffer cells, and ICAM expression in the aorta compared with that in DM mice. (A) STZDM-JNK1^-/-^ mice demonstrated a significant decrease of serum NO levels compared with that in DM mice. (B) STZDM-JNK1^-/-^ mice demonstrated a significant decrease of IL-1β expression in Kupffer cells compared with that in DM mice. Kupffer cells were purified from liver. The total RNAs were extracted from Kupffer cells using the Miniprep Purification Kit (GeneMark). Real-time polymerase chain reaction was performed with the SYBR Green PCR Master Mix and ABI PRISM 7700 Sequence Detection Systems (Applied Biosystems, Foster City, CA). (C) STZ-DM mice demonstrated a significant increase of pJNK expression of intestinal mucosa as compared with that in WT mice. (D) STZDM-JNK1^-/-^ mice demonstrated a significant decrease of ICAM expression of aorta as compared with that in DM mice. ICAM, intercellular adhesion molecule; JNK, c-Jun NH2-terminal kinase; Fmo, flavin mono-oxygenase; DM, diabetes mellitus. *, *P* < 0.05; **, *P* < 0.01; ***, < 0.001. n = 6/group.

To further analyze the role of pJNK1 in diabetes-induced serum NO levels, we examined the serum NO levels in STZDM-JNK1^-/-^ mice. We observed that STZDM-JNK1^-/-^ mice demonstrated a significant decrease in serum NO levels compared with that in DM mice (Fig 7A). These results further prove that the JNK pathway plays a critical role in diabetes-induced serum NO levels, and pJNK1 inhibition decreases these levels.

### STZDM-JNK1^-/-^ mice demonstrate decreased IL-1β expression in Kupffer cells

We have demonstrated that diabetes induced a significant increase in phospho-JNK1 protein expression in the intestinal mucosa. To examine whether pJNK1 inhibition could decrease diabetes-induced Kupffer cell activation, we analyzed the IL-1β expression in Kupffer cells in STZDM-JNK1^-/-^ mice. A significant decrease in IL-1β expression in Kupffer cells was observed in STZDM-JNK1^-/-^ mice compared with that in DM mice (Fig 7B). This finding indicates that the JNK pathway plays a critical role in diabetes-induced Kupffer cell activation, and pJNK1 inhibition decreases Kupffer cell activation induced by diabetes.

### Diabetes-induced pJNK expression in the intestinal mucosa

The involvement of pJNK in the intestinal mucosa in diabetes was examined by assessing the pJNK1 protein expression in the intestinal mucosa of STZ-DM mice. These mice demonstrated a significant increase in pJNK expression in the intestinal mucosa compared with that in WT mice (Fig 7C), which show that diabetes induces pJNK expression in the intestinal mucosa.

### STZDM-JNK1^-/-^ mice demonstrate a significant decrease in ICAM expression in the aorta compared with that in DM mice

We examined the role of pJNK1 in diabetes-induced ICAM expression in the aorta by analyzing the ICAM expression in the aorta of STZDM-JNK1^-/-^ mice. We found that STZDM-JNK1^-/-^ mice demonstrated a significant decrease in ICAM expression in the aorta compared with that in DM mice (Fig 7D). This result indicates that the JNK pathway plays a critical role in diabetes-induced ICAM expression in the aorta, and pJNK1 inhibition could decrease this expression.

### GF mice cohoused with STZDM mice demonstrate a significant increase in ICAM expression in the liver

**Fig 8:**
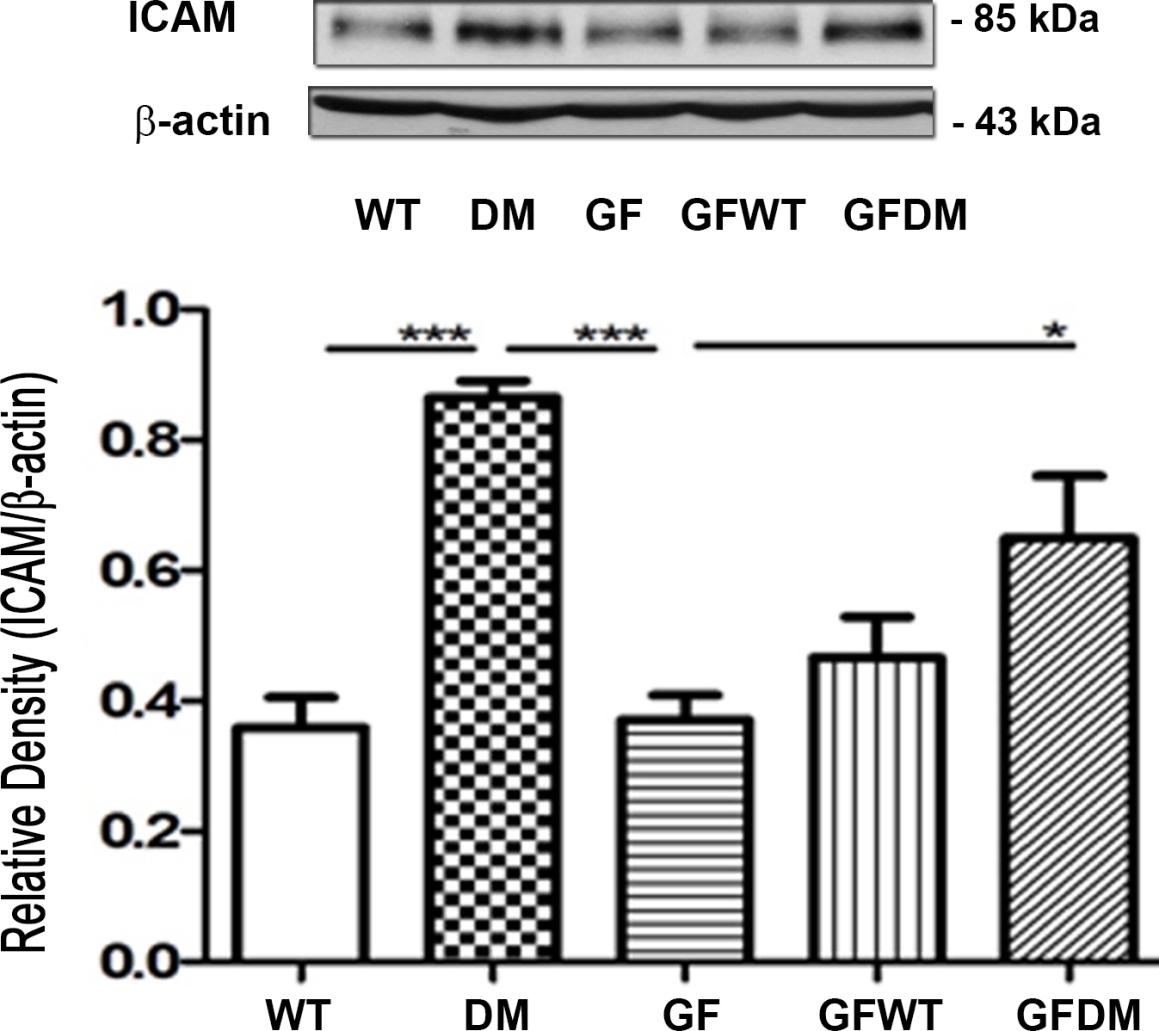
GF mice cohoused with DM mice demonstrated a significant increase of ICAM expression in the liver as compared with GF mice. GF mice cohoused with WT mice did not demonstrate a significant change of ICAM expression in the liver compared with that in GF mice. The ICAM protein expression in the intestinal mucosa was identified by mouse monoclonal antibodies (R&D Systems). A GF mouse was randomly chosen to cohouse with a wild-type mouse or STZDM mice and kept in a sterile lamina-flow hood for 2 weeks to examine whether the microbiota of the Ins2^Akita^ could change the ICAM expression of liver the GF mice. ICAM, intercellular adhesion molecule; GF, germ free mice; GFWT, germ free mice cohousing with WT mice; GFDM, germ free mice cohousing with STZ-DM mice. *, *P* < 0.05; **, *P* < 0.01; ***, < 0.001. n = 6/group.

To examine the role of commensal microflora in diabetes-induced ICAM expression in the liver, we analyzed the ICAM expression in the liver of GF mice and GF mice cohoused with STZDM mice. STZDM mice demonstrated a significant increase in ICAM expression in the liver compared with that in SPF mice. GF mice cohoused with STZDM mice demonstrated a significant increase in ICAM expression in the liver compared with that in GF mice. However, GF mice cohoused with WT mice did not demonstrate a significant change in ICAM expression in the liver compared with that in GF mice (Fig 8). These findings show that intestinal microflora play an important role in inducing ICAM expression in DM.

## Discussion

The relationship between commensal microflora and diabetes-induced endothelial inflammation and vascular impairment remains to be clarified. The findings of the present study suggest that diabetes induced Fmo3 and ICAM expression and possible vascular impairment through enteric dysbiosis-related JNK pathways in the host. Using 16S rRNA, we clearly showed that diabetes induced a significant increase in total bacteria and a decrease in *Lactobacillus* growth in the intestine. Next, we used prebiotic or probiotic feeding to selectively stimulate the growth of certain commensal bacteria such as *Lactobacillus*. FOS supplementation significantly increased *Lactobacillus* growth in the intestine of STZ-DM mice. Reversal of diabetes-induced enteric dysbiosis with FOS or dead *L*. *plantarum* feeding restored the diabetes-induced reduction of Reg3β protein expression in the intestinal mucosa. These results indicate that prebiotic or probiotic treatment reverses the diabetes-induced reduction of antibacterial protein expression in the intestinal mucosa. Moreover, FOS or dead *L*. *plantarum* feeding significantly decreased the diabetes-induced Fmo3 and ICAM expression in STZ-induced diabetic and Ins2^Akita^ mice. Our data suggest that diabetes induces enteric dysbiosis, Fmo3 expression in the liver, and ICAM expression in the aorta. Reversal of diabetes-induced enteric dybiosis with prebiotic or probiotic treatment significantly decreases the diabetes-induced Fmo3 and ICAM expression. Furthermore, GF mice cohoused with DM mice demonstrated a significant increase in ICAM expression in the liver compared with that in GF mice. Altogether, these results suggest that the intestinal microbiota play an important role in diabetes-induced Fmo3 expression in the liver and ICAM expression in the liver and aorta. Reversal of enteric dysbiosis with FOS or dead *L*. *plantarum* feeding increases ROS production in the intestine and decreases iNOS expression, NO production, Fmo3 expression and ICAM expression in diabetes. Using prebiotic or specific probiotic supplementation to reverse diabetes-induced enteric dysbiosis could be a novel therapeutic strategy to reverse diabetes-induced vascular impairment in patients with DM.

We next examined the mechanism of enteric dysbiosis-induced Fmo3 and ICAM expression in diabetes. Oxygen free radicals play an important role in gut epithelial damage, which may change the gut barrier function, facilitate BT and release of endotoxin [20]. At normal levels, nitric oxide (NO) is a major mediator of intestinal cell and barrier function [9]. However, when NO is present in excess, the result is barrier dysfunction [10]. Moreover, intestinal NO has been demonstrated to function as an inducer for the interorgan immune communication between the gut and the liver. It is generally accepted that DM impairs endothelial nitric oxide synthase (eNOS) activity and increases the production of reactive oxygen species (ROS), thus resulting in reduced NO bioavailability and the consequent pro-atherogenetic alterations (3). An overexpression of NO following the activation of nNOS, iNOS, and eNOS contribute to the pathogenic role in a few liver diseases resulting in portal hypertension [21]. The Kupffer cells, inflammatory cells, and recruited macrophages result in the production of cytokines and chemokines that cause prolonged inflammation and hepatocyte damage [22]. Both STZ-DM mice and Ins2^Akita^ mice demonstrated a significant increase in intestinal iNOS protein expression compared to that in control mice. In addition, increased serum NO levels and IL-1β and IL6 expression in Kupffer cells was subsequently detected in the diabetic mice. Interestingly, all these effects caused by the nitrosative stress were reversed after treating with prebiotic or dead *L*. *plantarum* supplementation, including IL-1β and IL6 expression in Kupffer cells and Fmo3 and ICAM expression in the diabetic mice. Our results suggest that diabetes induces serum NO levels through the enteric dysbiosis-induced iNOS protein expression in the intestinal mucosa. Moreover, diabetes decreases the production of ROS in the intestinal mucosa. These results suggest that DM decreases the production of ROS in the intestinal mucosa and thus induces iNOS activity as well as NO production. Our data suggest that enteric dysbiosis-induced reduction of ROS production and increase in iNOS and NO production play an important role in diabetes-induced activation of Kupffer cells and Fmo3 and ICAM expression in the liver. Reversal of diabetes-induced enteric dysbiosis with prebiotic or probiotic feeding could decrease pJNK and iNOS expression in the intestinal mucosa, serum NO levels, and Fmo3 and ICAM expression in the liver and aorta in diabetic patients.

We have previously demonstrated that thermal injury-induced peroxynitrite production and iNOS, ICAM-1, and VCAM-1 expression in the lung are mediated by the JNK signaling pathways [23]. Ins2^Akita^ mice and STZ-DM mice demonstrated a significant increase in pJNK expression in the intestinal mucosa. FOS feeding significantly decreased pJNK expression in the intestinal mucosa of Ins2^Akita^ mice. These results indicate that diabetes induces pJNK expression in the intestinal mucosa and FOS feeding could reverse it. To further examine the role of pJNK1 in diabetes-induced serum NO levels, we analyzed serum NO levels in Ins2^Akita^-JNK1^-/-^ and STZDM-JNK1^-/-^ mice. Both Ins2^Akita^-JNK1^-/-^ mice and STZDM-JNK1^-/-^ mice demonstrated a significant decrease in serum NO levels compared with those in Ins2^Akita^ mice and JNK1^-/-^DM mice, respectively. Moreover, both Ins2^Akita^-JNK1^-/-^ and STZDM-JNK1^-/-^ mice demonstrated a significant decrease in IL-1β expression in Kupffer cells compared with that in Ins2^Akita^ mice and STZDM mice, respectively. Ins2^Akita^-JNK1^-/-^ mice demonstrated a significant decrease in Fmo3 expression in the liver compared with that in Ins2^Akita^ mice. STZDM-JNK1^-/-^ mice demonstrated a significant decrease in ICAM expression in the aorta compared with that in STZDM mice. These results further prove that phospho-JNK plays a critical role in diabetes-induced serum NO levels and pJNK1 inhibition decreases diabetes-induced serum NO levels and Fmo3 and ICAM expression. Altogether, these data indicate that diabetes induces enteric dysbiosis and reduction in the production of ROS in the intestine and increases intestinal pJNK and iNOS expression in the intestinal mucosa as well as serum NO levels. Inhibition of pJNK1 decreases diabetes-induced serum NO levels, Fmo3 and ICAM expression in the liver, IL-1β production in Kupffer cells, and ICAM expression in the aorta. These results suggest that diabetes induces NO production and Fmo3 and ICAM expression through the pJNK pathways, and pJNK inhibition could reverse diabetes-induced NO production, Kupffer cell activation, and Fmo3 and ICAM expression. pJNK inhibition could be a useful therapeutic strategy to decrease diabetes-induced ICAM expression and vascular impairment in patients with diabetes.

**Fig 9:**
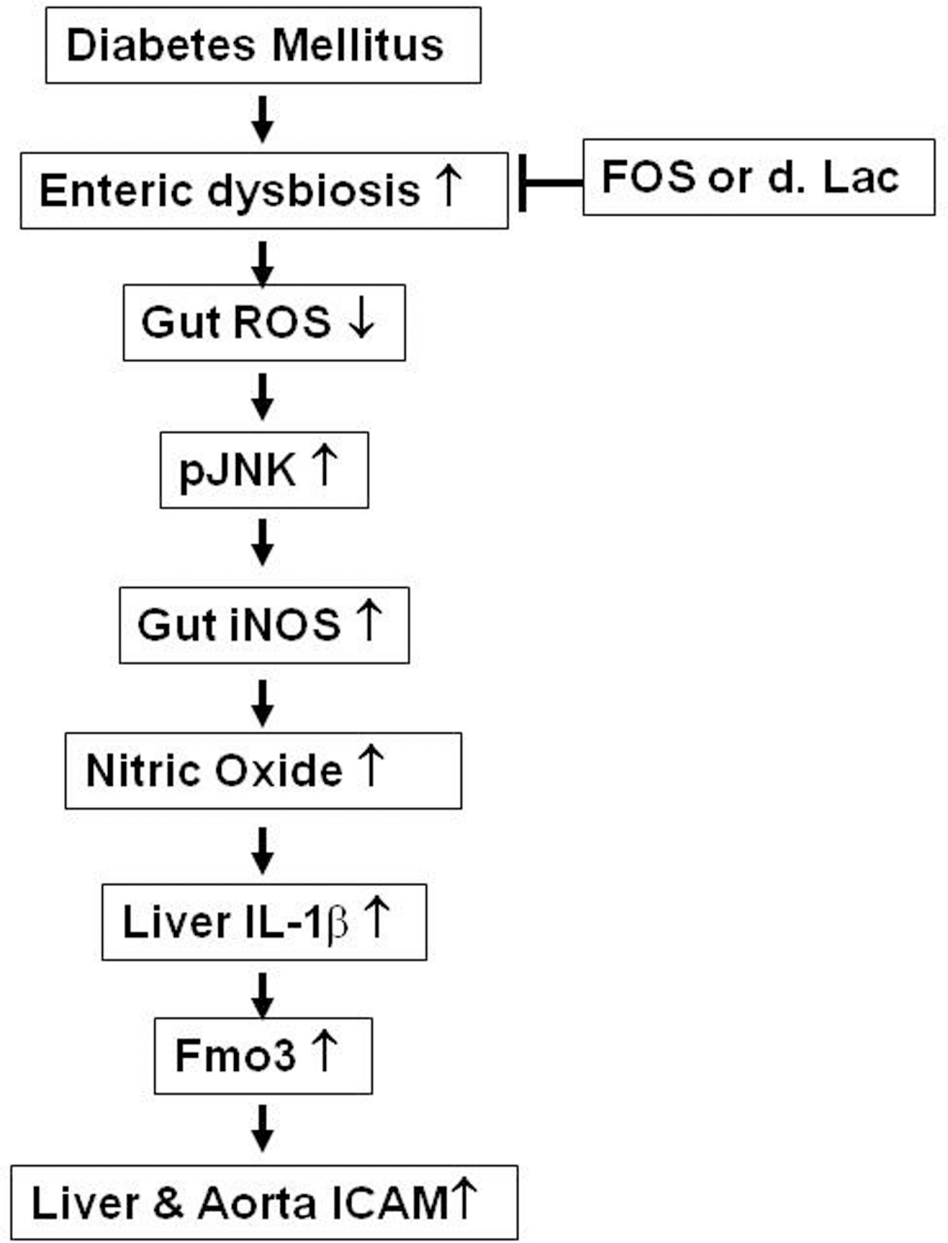
The regulatory mechanisms of enteric dysbiosis on Fmo3 and ICAM expression in diabetes. ICAM, intercellular adhesion molecule; Fmo, flavin mono-oxygenase; DM, diabetes mellitus; FOS, fructooligosaccharides; dLac, dead *L*. *plantarum*.

In conclusion, this study showed that diabetes induces Fmo3 and ICAM expression and possible vascular impairment through enteric dysbiosis-related JNK pathways. Diabetes decreases *Lactobacillus* growth and ROS levels in the intestine and induces iNOS and pJNK protein expression in the intestine, serum NO levels, activation of Kupffer cells, and Fmo3 and ICAM expression in the liver. Reversal of diabetes-induced enteric dysbiosis with prebiotic (FOS) or probiotic (dead *L*. *plantarum*) treatment increases intestinal ROS levels and decreases diabetes-induced iNOS expression in the intestine, Fmo3 expression in the liver, IL-1β expression in Kupffer cells, and ICAM expression in the aorta and liver. pJNK inhibition decreases serum NO levels, IL-1β expression in Kupffer cells, Fmo3 expression in the liver, and ICAM expression in the aorta (Fig 9).

## Supporting information

S1 Table Blood glucose levels and body weight in control, STZ-DM, STZ-DM+FOS, STZ-DM+dL.P. Mice. STZ, streptozotocin; DM, diabetes mellitus; FOS, fructooligosaccharides; dL.P., dead *L*. *plantarum*. ***P < 0.001 vs Control. n = 40/group.
(PDF)
