# Supplementary material for: Lactobacillus plantarum reverse diabetes-induced Fmo3 and ICAM expression in mice through enteric dysbiosis-related c-Jun NH2-terminal kinase pathways
Source: PLoS One. 2018 May 31;13(5):e0196511. doi: 10.1371/journal.pone.0196511 (PMC5978885; doi:10.1371/journal.pone.0196511)
Supplement: S1 Table — STZ, streptozotocin; DM, diabetes mellitus; FOS, fructooligosaccharides; dL.P., dead L. plantarum. ***P < 0.001 vs Control. n = 40/group. (PDF) [file pone.0196511.s001.pdf]

Supporting information

S1 Table

Blood glucose levels and body weight in Control, STZ-DM, STZ-DM+FOS, STZ-DM+dL.P. mice

Blood Glucose (mg/dl)			
**Control**	**STZ-DM**	**STZ-DM+FOS**	**STZ-DM+dL.P.**
**120±13**	**570±105*** **	**567±102*** **	**572±102*** **

Body Weight (g)			
**Control**	**STZ-DM**	**STZ-DM+FOS**	**STZ-DM+dL.P.**
**28.5±3**	**18.5±3*** **	**18.3±3*** **	**17.9±3*** **

STZ, streptozotocin; DM, diabetes mellitus; FOS, fructooligosaccharides; dL.P., dead *L. plantarum*. ***$P < 0.001$ vs Control. n = 40/group.
